# Inflammation-type dysbiosis of the oral microbiome associates with the duration of COVID-19 symptoms and long COVID

**DOI:** 10.1172/jci.insight.152346

**Published:** 2021-10-22

**Authors:** John P. Haran, Evan Bradley, Abigail L. Zeamer, Lindsey Cincotta, Marie-Claire Salive, Protiva Dutta, Shafik Mutaawe, Otuwe Anya, Mario Meza-Segura, Ann M. Moormann, Doyle V. Ward, Beth A. McCormick, Vanni Bucci

**Affiliations:** 1 Department of Emergency Medicine,; 2 Department of Microbiology and Physiological Systems,; 3 Program in Microbiome Dynamics, and; 4 Department of Medicine, University of Massachusetts Medical School, Worcester, Massachusetts, USA.

**Keywords:** COVID-19, Inflammation, Neurological disorders

## Abstract

In the COVID-19 pandemic, caused by SARS-CoV-2, many individuals experience prolonged symptoms, termed long-lasting COVID-19 symptoms (long COVID). Long COVID is thought to be linked to immune dysregulation due to harmful inflammation, with the exact causes being unknown. Given the role of the microbiome in mediating inflammation, we aimed to examine the relationship between the oral microbiome and the duration of long COVID symptoms. Tongue swabs were collected from patients presenting with COVID-19 symptoms. Confirmed infections were followed until resolution of all symptoms. Bacterial composition was determined by metagenomic sequencing. We used random forest modeling to identify microbiota and clinical covariates that are associated with long COVID symptoms. Of the patients followed, 63% developed ongoing symptomatic COVID-19 and 37% went on to long COVID. Patients with prolonged symptoms had significantly higher abundances of microbiota that induced inflammation, such as members of the genera *Prevotella* and *Veillonella*, which, of note, are species that produce LPS. The oral microbiome of patients with long COVID was similar to that of patients with chronic fatigue syndrome. Altogether, our findings suggest an association with the oral microbiome and long COVID, revealing the possibility that dysfunction of the oral microbiome may have contributed to this draining disease.

## Introduction

The oral cavity holds the second largest microbial community in the human body, after the gut, with over 1000 species of commensal bacteria residing therein (1). Dysbiosis or disrupted homeostasis caused by an imbalance in the microflora in the oral cavity has been linked to many other systemic inflammatory or infectious diseases (2). There is mounting evidence that links oral bacterial species to systemic diseases including pneumonia (1, 3, 4). Bacteria in the oral cavity may promote respiratory infections either directly via aspiration or indirectly by enzyme production that may hinder pathogen clearance, promote lung colonization, or alter respiratory epithelial immune responses (5).

SARS-CoV-2 is responsible for the current COVID-19 pandemic. This pandemic began in early 2020 and has caused over half a million deaths in the United States alone (6). Building upon the body of evidence that the microbiome plays a role in the regulation of innate and adaptive immunity to viral infections (7, 8), studies done early in the pandemic have demonstrated a connection between an altered gut microbiome and the severity of COVID-19 (9, 10). Additionally, among patients with COVID-19 there has been a large number of coinfection cases with organisms that originate from the oral cavity (11). Recently, decreased oral microbiome diversity and increased dysbiotic species abundances have been identified as predictive of COVID-19 (12). This has raised the possibility of using the oral microbiome to diagnose SARS-CoV-2 infection; however, studies linking the observed dysbiotic oral microbiota to disease outcomes have been lacking. Also lacking is evidence that this COVID-related microbiome, which occurs early in the disease process, is predictive of key outcomes such as symptom duration.

Most hospitalized patients have persistent, long-lasting symptoms that can take weeks to resolve (13) and negatively affect health-related quality of life (14). Symptoms persisting greater than 4 weeks after an acute infection are called ongoing symptomatic COVID-19, as characterized by The British National Institute for Health and Care Excellence (15). Symptoms lasting even longer, 8–12 weeks or greater (16), and symptoms characterized by fatigue, headache, dyspnea, and anosmia (17, 18) are termed long-lasting COVID-19 symptoms (long COVID). Long COVID does not currently have a strict definition (19). At the 10-week mark after SARS-CoV-2 infection, more than 50% of patients with long COVID suffer profound fatigue (20). Increasing age, body mass index, and female gender are known to associate with long COVID (16). It is currently unknown why most people recover fully within 2 to 3 weeks and others experience symptoms for weeks or months longer (21). There is evidence, however, of persistently perturbed inflammatory pathways long after the acute SARS-CoV-2 infection has subsided (22).

Given the emerging associations between the human microbiome and SARS-CoV-2 infection and the unknown driver for patients with COVID-19 suffering from long-lasting symptoms, we sought to explore if oral microbiome dysbiosis associates with ongoing symptoms among patients with COVID-19 after hospitalization. Accordingly, we enrolled a cohort of patients with COVID-19 who tested positive for SARS-CoV-2 infection by PCR from an emergency department in the United States, collected oral swabs early in the disease course, and followed them for 4-week and 10-week symptom resolution outcomes. We analyzed oral microbiome composition by shotgun metagenomic sequencing. Our findings uniquely describe how dysbiosis of the oral microbiome may have played a pivotal role in lengthening symptom duration, leading to the long COVID syndrome.

## Results

### Patient population.

**Figure 1:**
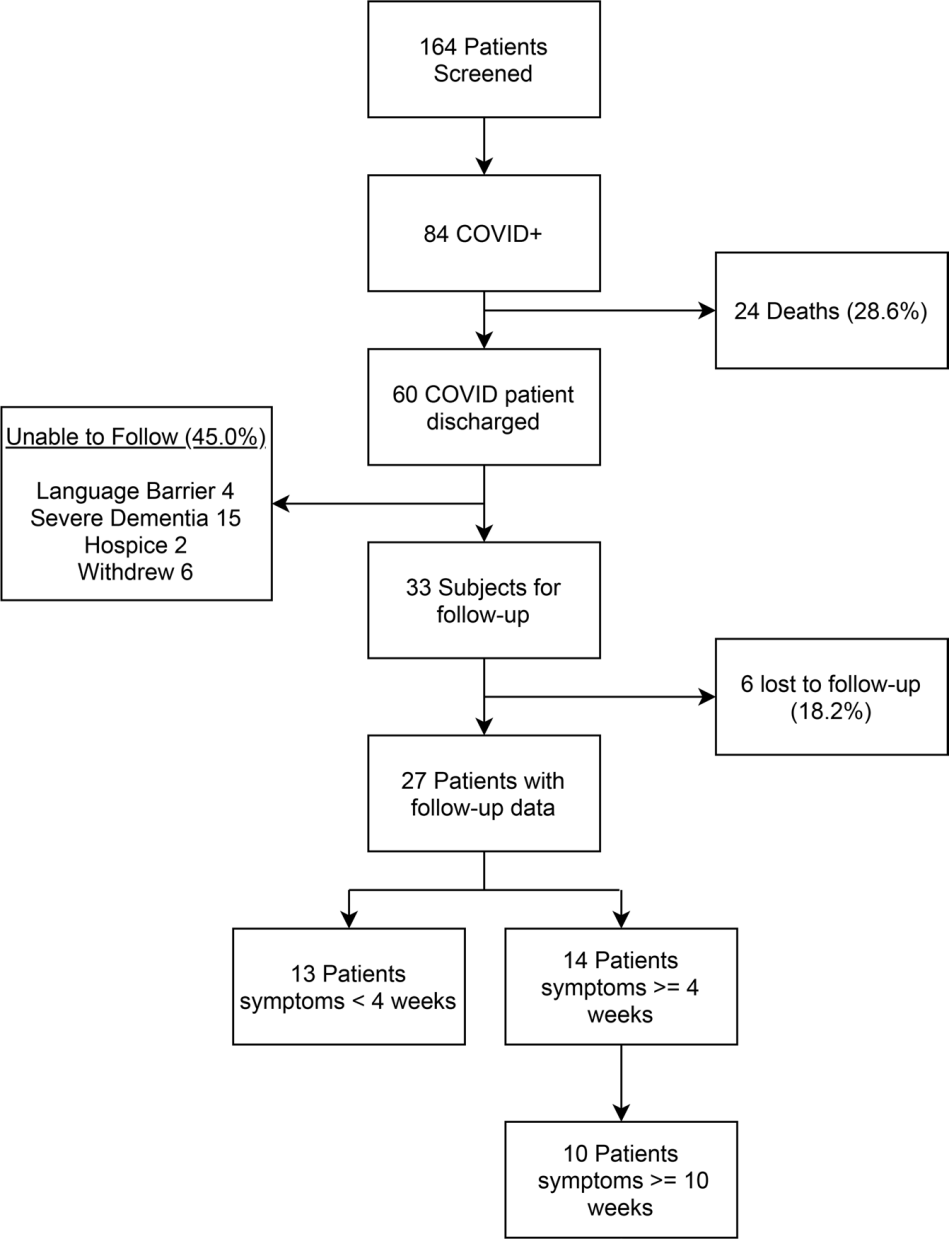
Study enrollment flow chart.

From a prospective sampling of 164 patients presenting with COVID-19 symptoms over a 9-month period, 84 (51.2%) tested positive by PCR for SARS-CoV-2. Of these patients, 27 were successfully contacted for follow-up at both 4 weeks and 10 weeks (Figure 1). The average age was 62.6 (SD 12.5) with 70.4% men, 66.7% White, 7.4% African American, and 25.9% Hispanic. Among the cohort for high-risk medical comorbidities, 16 (59.3%) had hypertension, 8 (29.6%) diabetes, and 5 (18.5%) chronic obstructive pulmonary disease. Neither the medical comorbidities nor the patients’ Charlson Comorbidity Index (CCI) scores differed by symptom duration outcome (Table 1). None of these patients lived in the same household. All of these patients were admitted to the hospital, with 4 (14.8%) admitted to the ICU. The average hospital length of stay was 8.3 days (SD 7.7), with 85.2% requiring oxygen and 25.9% getting advanced oxygen delivery by high flow or positive airway pressure. Two patients were intubated with an endotracheal tube.

### Symptom duration.

The average length of symptom duration was 45.8 days (SD 30.4), with 14 patients (51.9%) experiencing continuation of symptoms after 4 weeks from disease onset, and 10 patients (37.0%) experiencing symptoms longer than 10 weeks. The symptoms that lasted the longest were respiratory in nature (81.5% cough or short of breath) followed by fatigue (55.6%), gastrointestinal symptoms (14.8%), confusion or “brain fog” (22.2%), and ageusia or anosmia (14.8%). Brain fog is a symptom more recently linked to long COVID and characterized by a lack of clear memory or an ability to focus (23, 24). There were no significant differences in demographics, medical history, or hospital treatments among the 2 outcome categories (Table 1). However, among patients with symptoms lasting longer than 10 weeks, fatigue and brain fog were the most prominent symptoms that lasted the longest duration.

### Oral microbiome composition predicted ongoing symptomatic COVID-19.

**Figure 2:**
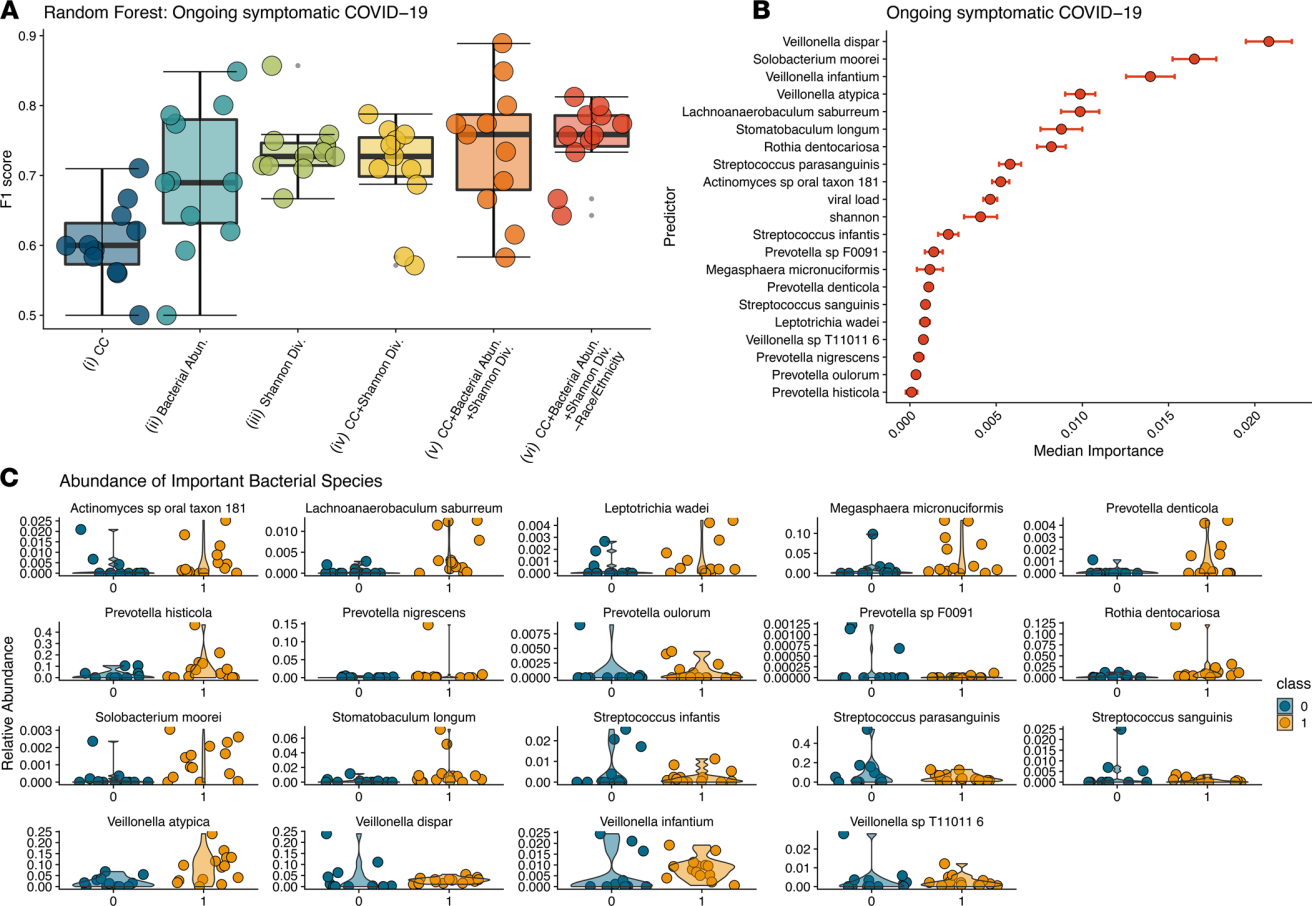
Bacterial abundances predict ongoing symptomatic COVID-19. RFC to identify predictors of ongoing symptomatic COVID-19 using 6 different combinations of data modalities. (**A**) F1 scores for the different RFC models trained on different sets of covariates. Box plot represents the median and IQR. (**B**) Ranking of forest predictors based on median permutated variable importance for the top-performing model. (**C**) Relative abundances for each bacteria found to be important in predicting ongoing symptomatic COVID-19 from the top-performing RFC model (on clinical data, microbiome species, and Shannon diversity). Violin plots showing the distribution of relative abundance for microbes in each patient with symptoms less than 4 weeks and 4 weeks or longer. 0 indicates “no” and 1 indicates “yes ongoing symptomatic COVID-19.” CC, clinical covariates; Abn, abundances; Div, diversity; RFC, forest classification modeling.

We set out to explore the associations of oral microbiome composition with the symptoms of ongoing symptomatic COVID-19. To do this we profiled the oral microbiome of subjects with acute COVID-19 infection using shotgun metagenomic sequencing (see Methods). Microbial species abundances were determined by running Metaphlan3 (25). We estimated microbiome α diversity by calculating Shannon diversity index (26). We started by applying unsupervised learning methods, such as principal coordinate analysis (PCoA) and t-distributed stochastic neighbor embedding (t-SNE) and, as expected, found that interindividual variability overwhelmingly accounted for the majority of the information in the data (Supplemental Figure 1; supplemental material available online with this article; https://doi.org/10.1172/jci.insight.152346DS1). PERMANOVA analysis on samples classified according to COVID-19 symptoms duration was not statistically significant (*P* < 0.05). We then applied random forest classification (RFC; refs. 27, 28) to identify microbiome and clinical features associated with ongoing disease. Feature selection was performed using the Boruta algorithm on 5-fold cross-validated data and then running RFC using the union of the selected Boruta features on the same 5-fold cross-validated data to estimate model performance (29). We compared classification accuracy for different models that were trained only on demographics and clinical data; only on microbiome species abundances; only on Shannon diversity; on demographics, clinical data, and Shannon diversity; on demographics, clinical data, microbiome species, and Shannon diversity; and on clinical data, microbiome species, and Shannon diversity (Figure 2A). Each model was run starting from 10 different random seeds to calculate appropriate performance statistics. The mean F1 score, the harmonic mean of precision and recall, was used to select the top-performing model for a given outcome. The best model — clinical data and microbiome species and Shannon diversity — performed with a mean F1 score of 0.751 (Figure 2A).

Specific microbial members had the greatest contribution to correctly classifying samples. We detected both bacterial and eukaryotic organisms in the oral microbiome analysis, with only bacteria demonstrating associations with the outcomes. We examined the 19 bacterial species, whose abundances were associated with ongoing symptomatic COVID-19, and 2 clinical covariates based on their median RFC-estimated permutated importance score over the 10 RFC pipeline iterations (Figure 2, B and C). The findings from the model indicate that both viral load and Shannon diversity were of moderate importance, whereas specific microbiome members contributed the most to correct sample prediction. In particular, 2 of the 3 top predictors (*Veillonella dispar* and *Veillonella infantium*) as well as 2 other species associated with ongoing symptomatic COVID-19 belong to the genus *Veillonella*. Members of this genus are gram-negative anaerobic coccus that can cause infection in humans (30). Specifically, *V*. *infantium* has been found in the bronchoalveolar lavage fluid of the patients with COVID-19, suggesting it is a significant coinfectious agent (31). Other pathobionts (organisms that can coexist or cause disease under certain circumstances), such as *Solobacterium moorei* (32, 33), *Streptococcus infantis* (34), and *Rothia dentocariosa* (35), were in higher abundances in patients with ongoing symptomatic COVID-19. Interestingly, *S*. *infantis* has been found to be enriched in fecal samples from patients with COVID-19 (9) and *R*. *dentocariosa* was predictive of SARS-CoV-2 presence in hospital rooms (36).

In addition to being implicated in coinfection, the *Veillonella* species is also known to produce a large amount of LPSs (37). Another pattern that emerges from these data is that the higher abundances of other LPS-producing species are predictive of ongoing symptomatic COVID-19. Five members of the *Prevotella* genus are positively associated with ongoing symptomatic COVID-19 in our analysis. *Prevotella* exhibits increased inflammatory properties (38) and has been thought to be a clinically important pathobiont involved in promoting chronic inflammation (39, 40). Other proinflammatory species such as *Leptotrichia wadei* (12) are also in higher abundances in patients with a longer symptom duration.

### Dysbiotic inflammatory-type oral microbiome associated with the development of long COVID syndrome.

**Figure 3:**
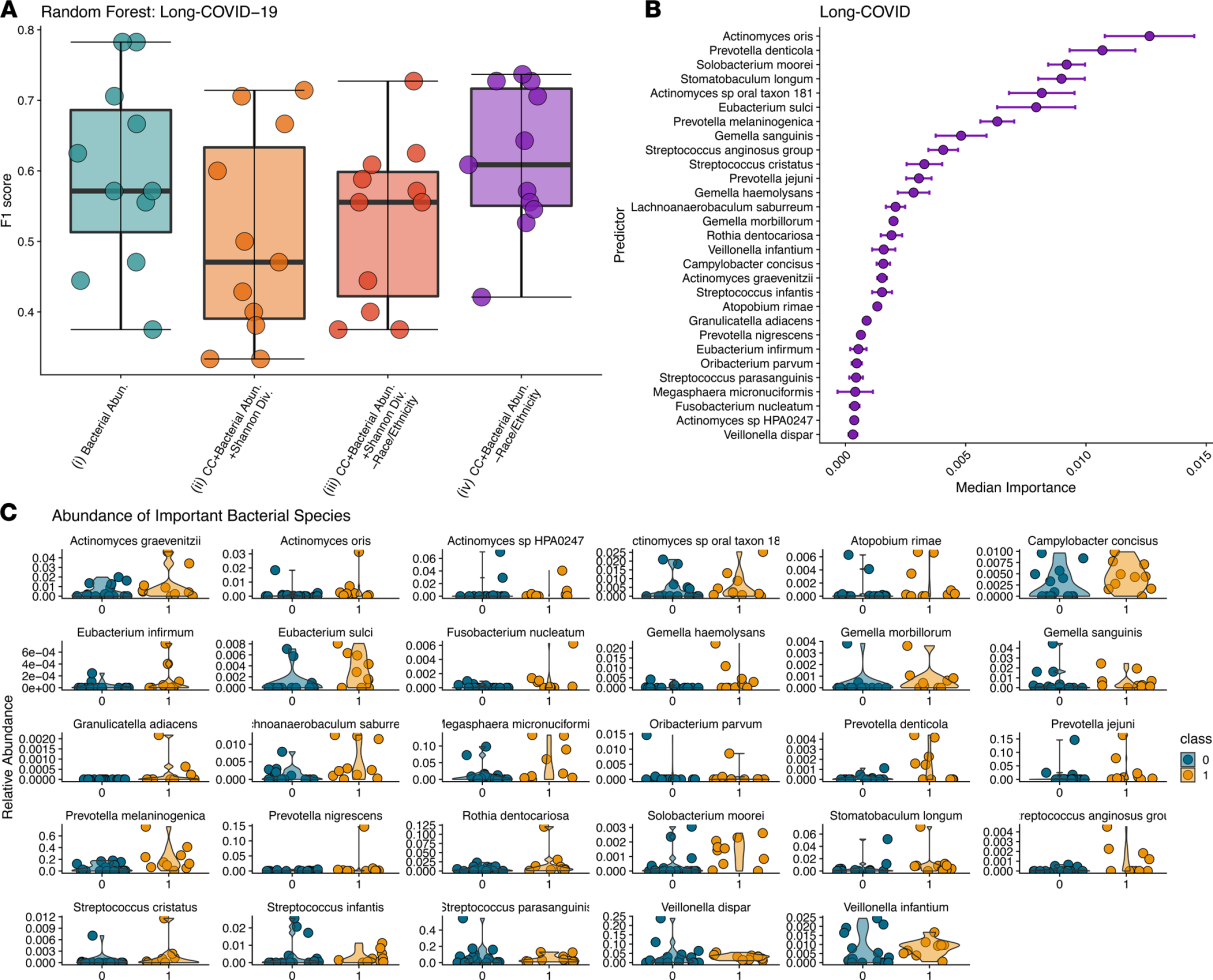
Bacterial abundances can predict long COVID. RFC modeling to predict long COVID. (**A**) F1 scores for all subsets of trainable RFC models. (**B**) Ranking of top 29 predictors associated with long COVID based on median permutated variable importance from the top-performing model (on demographics, clinical data, and Shannon diversity). (**C**) Relative abundances for each bacteria identified by model (on demographics, clinical data, and Shannon diversity) as important for predicting long COVID are presented as violin plots. Long COVID is presented as orange plots. CC, clinical covariates; Abn., abundances; Div., diversity. RFC, forest classification modeling.

We repeated our machine learning–based analysis described above to predict long COVID outcome from microbial abundance and clinical covariates. RFC was not able to capture any signal in the data for models that lacked microbiome information (i.e., only on demographics and clinical data; only on Shannon diversity; and on demographics, clinical data, and Shannon diversity; Figure 2A). The top-performing RFC for long COVID was the one trained on clinical data and microbiome species, resulting in an F1 score on 0.615 (Figure 3A). From the modeling, we identified 29 different bacterial species whose abundances were associated with long COVID (Figure 3, B and C). Similar to ongoing symptomatic COVID-19, multiple *Veillonella* species were associated with long COVID. Several of the top-predicting species (4 out of 29) belong to the genus *Actinomyces*. *Actinomyces* cause actinomycosis, a rare infectious disease in which bacteria can spread to the respiratory tract causing inflammation (41). As with ongoing symptomatic COVID-19, multiple *Prevotella* species (38) are associated with long COVID. *Prevotella* species are overrepresented in patients with COVID-19 and are thought to produce proteins that can promote SARS-CoV-2 infection and increase clinical severity of COVID-19 (42). Additional species known to cause infections such as the *S*. *anginosus* group bacterial species, which has been reported to be particularly important in the pathogenesis of respiratory infections (43), and *Gemella sanguinis,* which has been shown to cause bloodstream infections in patients with COVID-19 (44) were also found to be associated with long COVID.

### Inflammatory metabolic pathways associated with ongoing symptomatic COVID-19 and long COVID states.

**Figure 4:**
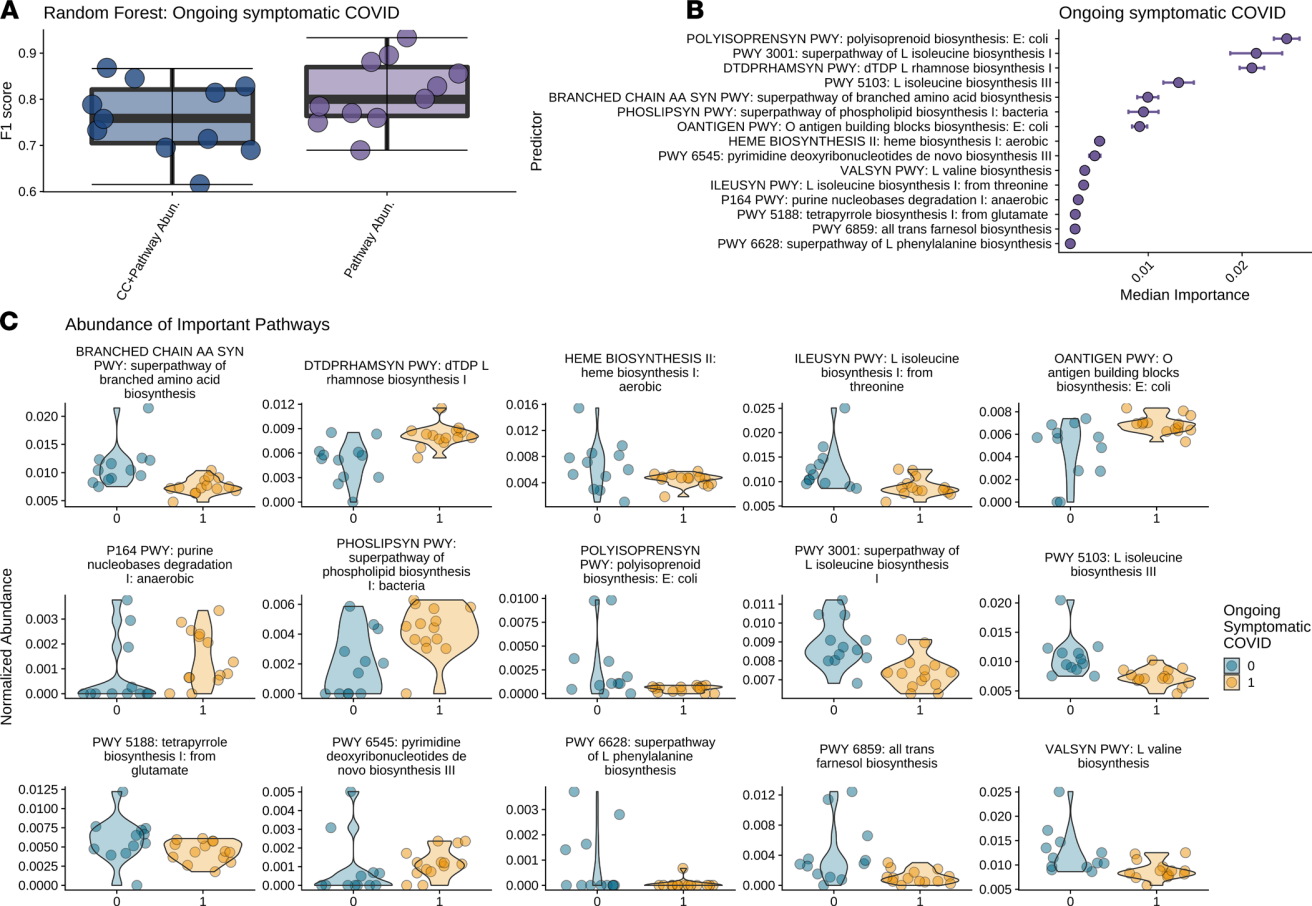
Bacterial metabolic pathways involving inflammation are significantly associated with ongoing symptomatic COVID-19. Results from RFC modeling to predict ongoing symptomatic COVID-19 and long COVID from HUMAnN3 pathway abundances. (**A**) F1 scores for demographics, clinical covariates, and pathway abundances and only on pathway abundances. (**B**) Ranking of forest predictors based on median permutated variable importance from the top-performing model, pathways only, for each outcome. (**C**) Relative pathway abundances for each pathway found to be important in predicting ongoing symptomatic COVID-19 and long COVID, respectively, by RFC modeling using only pathway abundances. We report violin plots showing the distribution of the relative abundance of pathways in patients with symptoms less than 4 weeks (blue) and 4 weeks or longer (yellow). RFC, forest classification modeling.

**Figure 5:**
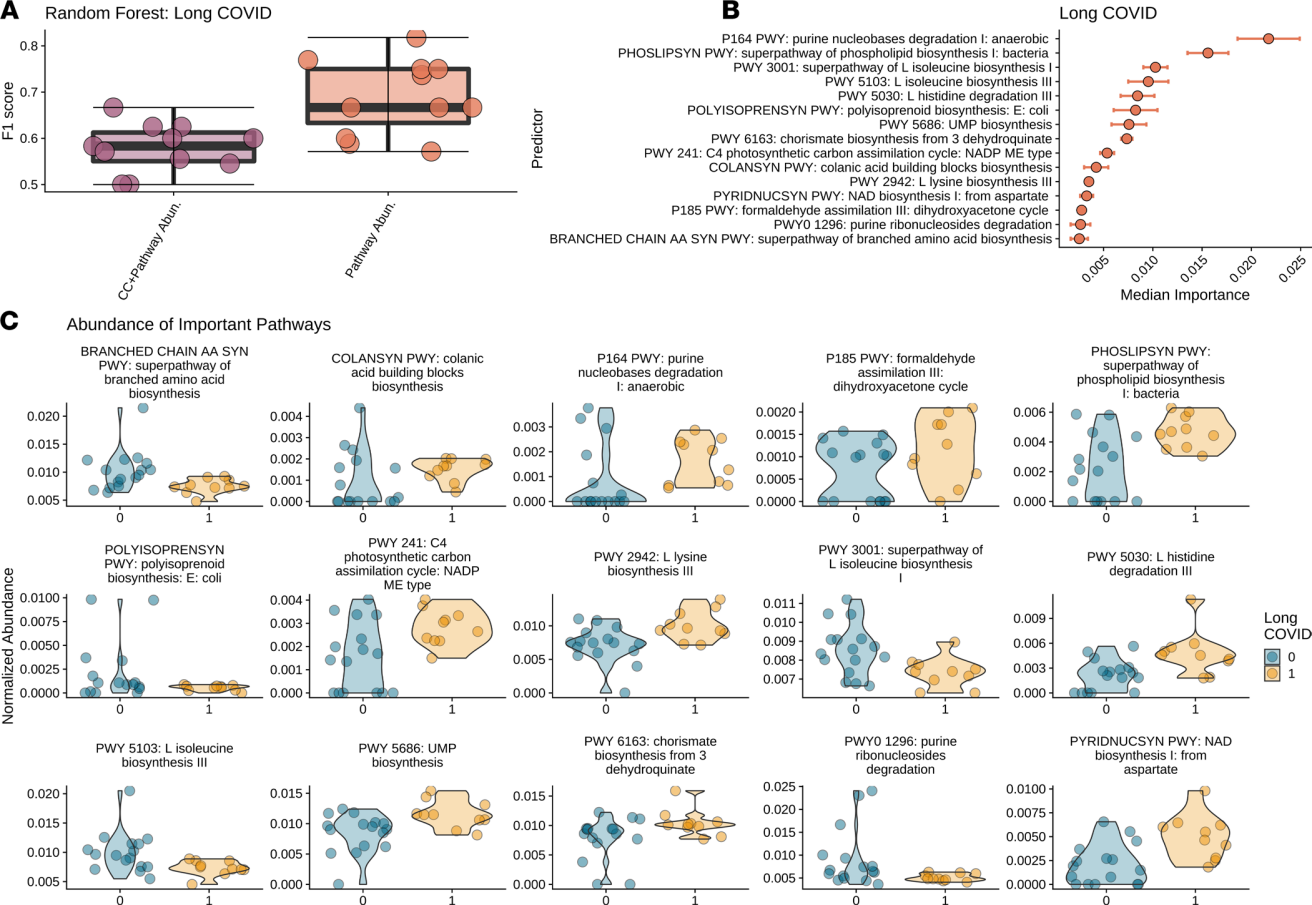
Bacterial metabolic pathways involving inflammation are significantly associated with long COVID. Results from RFC modeling to predict ongoing symptomatic COVID-19 and long COVID from HUMAnN3 pathway abundances. (**A**) F1 scores for (i) demographics, clinical covariates, and pathway abundances and only on pathway abundances. (**B**) Ranking of forest predictors based on median permutated variable importance from the top-performing model, pathways only, for each outcome. (**C**) Relative pathway abundances for each pathway found to be important in predicting long COVID, respectively, by RFC modeling using only pathway abundances. We report violin plots showing the distribution of the relative abundance of pathways in patients with symptoms less than 10 weeks (blue) and 10 weeks or longer (yellow). RFC, forest classification modeling.

Building upon the taxonomy analysis, we explored the metabolic pathways and their association with ongoing symptomatic COVID-19 and long COVID states using HUMAnN3 (45). For each outcome we again performed RFC analysis and compared classification accuracy for different trained models: demographics, clinical data, and relative pathway abundances and only relative pathway abundances. For both patients with ongoing symptomatic COVID-19 and patients with long COVID, the top-performing model was (ii) only relative pathway abundances, producing an F1 score of 0.814 and 0.689, respectively (Figure 4A and Figure 5A). We identified greater than 40 metabolic gene pathways whose abundances were associated with both ongoing symptomatic and long COVID (Figure 4B and Figure 5B). The top 15 predictors indicate a striking proinflammatory pattern.

For ongoing symptomatic COVID-19, there are 5 pathways involved in the biosynthesis of branched-chain amino acids that are reduced in patients with longer symptoms (Figure 4, B and C). These include the superpathway of L-isoleucine I (MetaCyc PWY-3001), L-isoleucine biosynthesis III (PWY-5103), superpathway of branched amino acids (BRANCHED-CHAIN-AA-SYN-PWY), L-valine (VALSYN-PWY), and L-isoleucine (ILEUSYN-PWY) biosynthesis pathways (ref. 46; Figure 4C). Branched amino acids have been shown to act as antiinflammatory agents (47, 48) with orally administered L-isoleucine and L-leucine exhibiting antiinflammatory activities (49). Four of 15 of the top pathways involve synthesis of molecules with antiinflammatory effects and are lower in patients with ongoing symptomatic COVID-19. These include the top predictor, Polyisoprenoid (50), whose biosynthesis has also been identified as significantly decreased in inflammatory conditions such as Crohn’s disease (51). Tetrapyrrole (52) and farnesol (53) also have antiinflammatory effects. Conversely, 3 pathways for biosynthesis of proinflammatory molecules are increased in patients with ongoing symptomatic COVID-19: dTDP-L-rhamnose (DTDPRHAMSYN-PWY; ref. 54), pyrimidine (PWY-6545; ref. 55), and purine (P164 PWY; ref. 56) deoxyribonucleotides. Finally, both O-antigen building block biosynthesis (OANTIGEN-PWY), an important step in the LPS biosynthetic pathway (57), and the superpathway of phospholipid biosynthesis (PHOSLIPSYN-PWY), important in LPS production (58, 59), are higher among patients with ongoing symptomatic COVID-19. Similar patterns emerge with the long COVID analysis, which share 6 predictors with the ongoing symptomatic COVID-19 analysis.

Proinflammatory molecule synthesis was higher among patients with long COVID relative to those without as well as reduced branch-chain amino acid and antiinflammatory molecule biosynthesis (Figure 5C). Additional proinflammatory molecule biosynthesis is noted, with chorismite (PWY-6163; ref. 60), colanic acid (COLANSYN-PWY; ref. 61), and NAD biosynthesis (PWY-241; ref. 62) all being higher among the patients with long COVID.

## Discussion

Many patients recovering from SARS-CoV-2 infection have symptoms that last long after the acute infection has run its course and our study highlights this same phenomenon. Over one third of our cohort had symptoms lasting longer than 10 weeks and thus entered the long COVID stage. Fatigue and “brain fog” were the longer lasting, most prominent symptoms among these patients. In an attempt to better understand both patients with ongoing symptomatic COVID-19 and patients with long COVID, we investigated potential clinical and microbiome associations with these disorders. Our modeling identified (a) microbial associations that are known to promote inflammation via LPS production or other mechanisms, (b) reduction of antiinflammatory metabolic pathways, (c) pathobionts known to cause pulmonary infections, and (d) microbiota previously shown to have associations with COVID-19. Thus, our work begins to shed light on the hypothesis that the oral microbiome composition may influence the duration of COVID-19 symptoms.

### Patients with longer COVID-19 symptoms had dysbiotic, inflammatory-type oral microbiome.

The oral microbiome has been shown to closely associate with SARS-CoV-2 coinfections in the lungs (11) and the oral–lung aspiration axis is a key factor leading to many respiratory infectious processes (63). We hypothesized that the oral microbiome might associate with the duration of postacute infection symptoms presented in ongoing symptomatic COVID-19 and long COVID states (64). Our findings extend previous work demonstrating how specific members of the genera *Prevotella* and *Veillonella* were distinctive in the oral microbiota of patients with COVID-19 (65). *Prevotella* species have been overrepresented in COVID-19 patient populations (42), whereas members of both the *Prevotella* and the *Veillonella* genera have been found in the bronchoalveolar lavage fluid of the patients with COVID-19 (31). Members of the *Prevotella* genus are thought to produce proteins that can promote SARS-CoV-2 infection and increase clinical severity of COVID-19 (42) and have previously been tied to systemic diseases, including low-grade systemic inflammation (38). The increased abundances of these 2 genera on the tongue have also been associated with an increased risk of death due to pneumonia in older, frail patients (66, 67). Finally, both genera induce inflammatory responses. *Veillonella* species have shown a strong capacity to induce IL-6 (68), whereas *Prevotella* strains primarily activate TLR-2 and enhance the expression of inflammatory cytokines, including IL-23 and IL-1 (69, 70). Other proinflammatory microbiota were identified in our analysis that also associated with longer disease symptoms such as *L*. *wadei* (12), *S*. *moorei* (71), and multiple *Actinomyces* species (41).

Metabolic pathways associated with the production of proinflammatory molecules were increased in abundance, whereas pathways associated with the production of antiinflammatory molecules were decreased in patients presenting with ongoing and long COVID symptoms. One of the top predictors, and thus demonstrating the strongest association in our data with both ongoing symptomatic COVID-19 and long COVID, was polyisoprenoid biosynthesis. Polyisoprenoid expresses antiinflammatory activity (50) and is significantly decreased in inflammatory conditions such as Crohn’s disease (51). Among the top predictors in our analysis was a reduced abundance of genes involved in the production of branched amino acids. Branched amino acids have long been shown to act as antiinflammatory agents (47, 48). Evidence is accumulating to support the hypothesis that systemic chronic inflammation contributes to the symptomatic progression to long COVID (22, 72). Given that changes in the microbiome composition can result in chronic inflammation and metabolic dysfunction (73), it is possible that the proinflammatory microbiome profiles we observe here could have played a pivotal role in this disease process.

### LPS-producing bacteria may have promoted inflammation and have driven COVID-19 symptom duration.

LPSs is an outer membrane component of gram-negative bacteria and can also be released in vesicles (74). Vesicle-associated LPS can have proinflammatory effects on host immune systems (75). Microbiome-derived LPS causes systemic inflammation (76, 77) and can even induce cognitive impairment and neuroinflammation (78, 79). Increases in LPS-producing bacteria, such as *Leptotrichia*, have been demonstrated in the oral cavity of patients with COVID-19 and are thought to be involved in the inflammatory response (12). Our analysis reveals higher abundances of many LPS-producing bacteria in patients with longer lasting symptoms. For example, *Veillonella* species, known to produce large amounts of LPSs (37), are present in increased abundances in our patients with COVID-19 with longer lasting symptoms. Increases in species such as *V*. *dispar*, *V*. *infantium*, and *V*. *atypica* are top predictors of ongoing symptomatic COVID-19, whereas *V*. *infantium* is found in higher abundances among patients with long COVID. Other LPS-producing species such as *L*. *wadei* (12) and *M*. *micronuciformis* (80) are also found to be in increased abundances. Additionally, our metabolic pathway analysis revealed an association with important steps in LPS biosynthesis and ongoing symptomatic COVID-19 and long COVID states. It is possible that LPS production may be a marker of other risk factors rather than a direct causal contributor. This would be critical to investigate in future work; however, this evidence points toward the important association of inflammation and long symptom disease states.

### Myalgic encephalomyelitis/chronic fatigue syndrome linking to long-term COVID-19 symptoms through oral microbiome dysbiosis.

There has been a growing concern that patients with COVID-19 with long-term sequelae resemble patients with myalgic encephalomyelitis/chronic fatigue syndrome (ME/CFS; ref. 81). These 2 conditions share some of the same symptoms, especially fatigue and cognitive impairment (17, 82). ME/CFS is a condition characterized by chronic fatigue, lasting at least 6 months, that impairs one’s ability to perform daily activities and typically has additional impairments in memory and concentration (83). This syndrome is also closely linked to chronic inflammation as the driver of these patients’ symptoms (84). The link to long-term symptoms is not unique to COVID-19 because patients with both SARS-CoV-1 and Middle East respiratory syndrome have also suffered from long-term sequelae in the previous epidemics (85).

ME/CFS has been hypothesized to be linked to infectious agents and microbiome dysbiosis has specifically been described in this syndrome through either the presence of pathobionts or microbial species that promote chronic inflammation (86). The gut microbiome has been shown to have reduced diversity and altered composition in patients with ME/CFS (87), and viral-induced microbiome changes are also thought to play a pivotal role (88). Clinical trials targeting the gut microbiome have shown promise in treating ME/CFS (89). Interestingly, patients with ME/CFS have been shown to have altered dysbiotic oral microbiomes characterized by increased abundances in the genera *Leptotrichia*, *Prevotella*, and *Fusobacterium* (90). Using whole genome sequencing, we have shown many species belonging to these genera are increased abundance in both patients with ongoing symptomatic COVID-19 and patients with long COVID. Specifically, top-predicting species *L*. *wadei*, *P*. *sp F0091*, *P*. *denticola*, *P*. *nigrescens*, *P*. *histicola*, and *P*. *oulorum* in the ongoing symptomatic COVID-19 group and *P*. *denticola*, P. *melaninogenica*, *P*. *jejuni*, *P*. *nigrescens*, and *F*. *nucleatum* in the long COVID group were all present in higher abundances in patients suffering from longer lasting symptoms. These finding add intriguing evidence of a possible link between patients with ME/CFS and patients with COVID-19 suffering from longer lasting symptoms related to inflammation in the oral microbiome.

### Strengths and limitations.

This study has several notable strengths and limitations. This study is limited in the number of patients enrolled and followed for symptom duration outcomes. A more robust cohort would allow deeper investigation of preexisting medical conditions and medications that might shape the oral microbiome composition. Larger cohorts would also include a more diverse patient set involving those treated as outpatients and more intensive care unit admissions. Generalization of our findings would need to be performed in a more diverse patient population. This limitation is balanced by our application of whole genome sequencing, which provides greater resolution than 16S rRNA gene sequencing used in many of the previous microbiome investigations (91). We also applied RFC which enable us to include both clinical and microbiome data in our modeling (27, 28). This modeling approach has significant advantages compared with traditional classification techniques. Because it is agnostic to model structure (e.g., nonparametric regression), it does not need to meet common assumptions underlying classical regression techniques, and is able to intrinsically perform permutated ranked feature selection (29). We also have the advantage of collecting samples at the time of diagnosis before medical treatments that may alter the microbiome composition.

### Conclusions.

In conclusion, the oral microbiome of patients with prolonged symptoms falling under the ongoing symptomatic COVID-19 or long COVID states demonstrated a dysbiotic pattern of increased pathobionts, an increase in inflammation-inducing and LPS-producing microbiota, and a reduction of metabolic pathways known to have antiinflammatory properties. Although this work needs further validation, it supports the tenet that the microbiome may have played a role in prolonging symptom duration among COVID-19 through promotion of inflammation. The microbiome may therefore hold the key to better understanding the postinfection prolonged syndromes now facing patients after they recover from acute infection and provide a way to predict and subsequently act upon and prevent the development of long COVID.

## Methods

### Study setting and population.

This prospective cohort consists of patients presenting to an emergency department located in central Massachusetts from April 2020 through February 2021. We enrolled patients who presented with symptoms consistent with a COVID-19 infection, but we analyzed only those who tested positive by PCR for SARS-CoV-2 and could contact for follow-up. We defined symptoms of COVID-19 based on the CDC guidelines (92).

### Data collection.

We collected baseline factors that included demographics, medical history, and presenting disease duration, and symptomatology. Comorbidity was assessed at baseline using the CCI, a widely used instrument designed to measure the burden of medical diseases and predict mortality (93). Patients were then followed through their hospital course for treatment types and length of stay. After discharge from the hospital, subsequent healthcare visits were recorded through the medical record. Patients were contacted by phone after 4 weeks of total symptoms after discharge and then again a second time if they were experiencing ongoing symptoms, after 10 weeks. Patients were categorized as symptoms lasting longer than 4 weeks and symptoms lasting longer than 10 weeks for analysis. Patients were also queried as to the type of symptoms that lasted the longest. Patients were excluded from follow-up if they died, were unable to communicate in English, had severe dementia, were in hospice, or withdrew themselves from the study.

### Sample collection and processing.

Oropharyngeal samples were collected using OMNIgene•ORAL collection kits (OMR-120, DNAgenotek). Briefly, the posterior oropharynx was swabbed for 30 seconds and then the swab was inserted into a tube with a DNA/RNA stabilization buffer. Samples were heated to 65^o^C–70^o^C for 1 hour to inactivate SARS-CoV-2 (94) and stored frozen. Nucleic acids were extracted by first thawing samples and then treating with 5ul Proteinase K (P8107S, New England Biolabs) for 2 hours at 50^o^C. DNA and RNA was then extracted using ZymoBIOMICS DNA/RNA Miniprep Kits (R2002, Zymo Research) as per the manufacture’s protocol.

### Sequence processing and analysis.

Metagenomic DNA sequencing libraries were constructed using the Nextera XT DNA Library Prep Kit (FC-131-1096, Illumina) and sequenced on a NextSeq500 Sequencing System as 2 × 150 nucleotide paired-end reads. Shotgun metagenomic reads were first trimmed and quality filtered to remove sequencing adapters and host contamination using Trimmomatic (95) and Bowtie2 (96), respectively, as part of the KneadData pipeline (https://bitbucket.org/biobakery/kneaddata). As in our previous work (28, 97), metagenomic data were profiled for microbial taxonomic abundances and microbial metabolic pathways using Metaphlan3 (25) and HUMAnN3 (45), respectively. The total number of microbial and contaminant reads recovered as presented in Supplemental Table 1.

### SARS-CoV-2 viral load quantification.

PCR was performed using the ViiA 7 Real-Time PCR System (Applied Biosystems) and the GoTaq Probe 1-Step RT-qPCR System (Promega, A6120). The primer-probe set N1 (2019-nCoV_N1-F: 5′-GACCCCAAAATCAGCGAAAT-3′; 2019-nCoV_N1-R: 5′-TCTGGTTACTGCCAGTTGAATCTG-3′; 2019-nCoV_N1-P: 5′-FAM-ACCCCGCATTACGTTTGGACC-BHQ1-3′) designed by the CDC were obtained from Integrated DNA Technologies (10006713) and used at concentrations of 500 nM and 125 nM, respectively (98). Eluted RNA (5 μl) were used to prepare 20 μl PCR reactions. Cycling conditions were as indicated by the CDC: 45°C for 15 minutes, 95°C for 2 minutes, followed by 45 cycles of 95°C for 3 seconds and 55°C for 30 seconds (98). Cycle threshold values were converted into viral RNA copies based on a standard curve prepared from 4-fold serial dilutions of known quantities (1.0 × 106 to 2.44 × 102 viral copies) of a SARS-CoV-2_N positive control plasmid (10006625, Integrated DNA Technologies). The lower limit threshold for positive detection in our study was 244 viral copies per reaction. Viral load was calculated as the number of genome copies per milliliter of transport media to resuspend tongue swabs. The assay was run in triplicate for each sample and 3 nontemplate wells were included as negative controls.

### Data availability.

Data relating to the metagenomic sequencing that support the findings of this study have been uploaded to the NCBI BioProject (https://www.ncbi.nlm.nih.gov/bioproject/) and are available for download via accession PRJNA735193 under the title “Oral Microbiome associated with Coronavirus disease 2019 (COVID-19).”

### Statistics.

To determine similarity in oral microbiome samples among the patients with COVID-19 and to associate microbiome features to duration of symptom outcomes, we started by performing traditional unsupervised correspondence analysis (PCoA and t-SNE). Because most of the signal from the unsupervised analysis was accounted by interindividual variability, we then decided to run supervised machine learning models. We built a RFC pipeline to predict either ongoing symptomatic COVID-19 or long COVID from a given data subset. One sample failed the sequencing run, and thus 26 samples were included in our modeling. The first step of our pipeline used the feature selection algorithm Boruta on 5-fold cross-validated data to estimate model performance (29). The permutated variable importance from each RFC was also calculated. Each model was run starting from 10 different random seeds to calculate performance metrics. F1 score, the harmonic mean of precision and accuracy, was used to select the top-performing model for each outcome. *P* values of less than 0.05 were considered significant.

### Study approval.

This prospective cohort study was approved by the IRB at the University of Massachusetts Medical School. Written informed consent was received from all study participants prior to inclusion in the study.

## Author contributions

JPH, BAM, AM, and EB conceived and led the study. JPH, EB, and PD supervised the conduct of the study and data collection. PD, MCS, SM, and OA managed the clinical data, including quality control. LC OA, and MMS handled the sample collection and storage. DVW managed sample extraction and sequencing and performed metagenomic profiling. ALZ and VB provided statistical advice on study design and analyzed the data. JPH and EB wrote the manuscript with input from all authors. JPH composed the first draft of the majority of the manuscript and was responsible for incorporation of all authors edits. Accordingly, JPH was assigned the first author slot.

## Supplementary Material

Supplemental table 1

## Figures and Tables

**Table 1:**
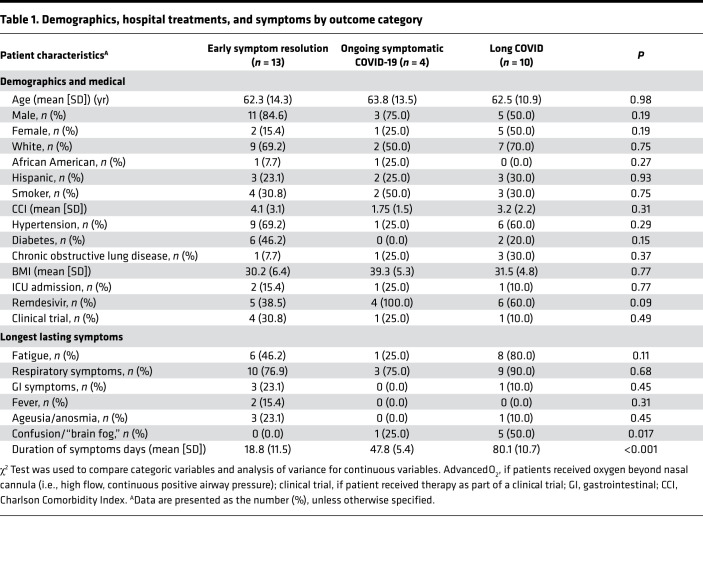
Demographics, hospital treatments, and symptoms by outcome category

## Version 1 08/17/2021

In-Press Preview

## Version 2 10/22/2021

Electronic publication
