# Supplementary material for: Inflammation-type dysbiosis of the oral microbiome associates with the duration of COVID-19 symptoms and long COVID
Source: JCI Insight. 2021 Oct 22;6(20):e152346. doi: 10.1172/jci.insight.152346 (PMC8564890; doi:10.1172/jci.insight.152346)
Supplement: Supplemental table 1 [file jciinsight-6-152346-s090.pdf]

Supplemental Table 1: Sequencing reads from each sample

Sample ID	Total Reads	Total Contam	R1 Metag Paired	R1 Metag Unpaired	R2 Metag Paired	R2 Metag Unpaired	R1 Contam Paired	R1 Contam Unpaired	R2 Contam Paired	R2 Contam Unpaired
CHC10103	1,012,891	34,424,360	335,984	167,903	335,984	173,020	16,559,216	901,443	16,559,216	404,485
CHC10104	20,236,681	14,525,368	9,764,560	484,302	9,764,560	223,259	6,962,721	376,218	6,962,721	223,708
CHC10105	853,438	28,710,414	311,762	117,616	311,762	112,298	13,620,984	940,516	13,620,984	527,930
CHC10106	3,362,861	63,280,232	1,394,025	310,029	1,394,025	264,782	28,798,434	3,459,553	28,798,434	2,223,811
CHC10115	5,111,452	46,164,679	2,222,951	374,040	2,222,951	291,510	20,888,308	2,601,437	20,888,308	1,786,626
SCVD030	1,336,383	17,076,965	544,760	145,449	544,760	101,414	7,701,798	1,092,277	7,701,798	581,092
SCVD048	1,949,734	37,905,329	812,344	180,460	812,344	144,586	17,474,757	1,729,102	17,474,757	1,226,713
SCVD079	1,032,373	43,237,734	367,341	164,791	367,341	132,900	19,988,829	1,932,160	19,988,829	1,327,916
SCVD128	6,700,616	23,192,009	2,988,588	418,950	2,988,588	304,490	10,553,244	1,253,769	10,553,244	831,752
SCVD129	4,156,351	36,833,053	1,900,706	198,364	1,900,706	156,575	17,771,266	890,742	17,771,266	399,779
SCVD131	871,703	30,864,328	337,272	103,252	337,272	93,907	14,543,851	1,110,319	14,543,851	666,307
SCVD132	3,095,655	18,663,276	1,367,498	207,700	1,367,498	152,959	8,562,504	934,888	8,562,504	603,380
SCVD135	3,690,522	6,047,358	1,630,896	253,527	1,630,896	175,203	2,691,454	393,247	2,691,454	271,203
SCVD138	7,158,271	30,493,962	3,352,217	271,875	3,352,217	181,962	14,608,038	858,958	14,608,038	418,928
SCVD141	7,951,136	14,881,428	3,637,656	385,022	3,637,656	290,802	6,802,068	726,752	6,802,068	550,540
SCVD145	2,618,272	14,660,326	1,165,040	170,484	1,165,040	117,708	6,646,978	827,718	6,646,978	538,652
SCVD149	12,357,568	14,995,355	5,669,542	650,081	5,669,542	368,403	6,868,207	800,216	6,868,207	458,725
SCVD151	21,701,748	18,385,572	10,179,762	826,585	10,179,762	515,639	8,639,698	696,134	8,639,698	410,042
SCVD155	11,697,572	13,303,612	5,344,131	610,189	5,344,131	399,121	6,062,729	717,477	6,062,729	460,677
SCVD158	5,013,754	28,788,410	2,351,166	179,579	2,351,166	131,843	13,909,974	648,000	13,909,974	320,462
SCVD159	13,269,437	16,955,886	6,132,040	601,723	6,132,040	403,634	7,806,357	798,515	7,806,357	544,657
SCVD160	12,140,620	8,721,154	5,593,143	587,854	5,593,143	366,480	3,983,447	460,771	3,983,447	293,489
SCVD166	1,153,447	14,342,157	485,810	101,079	485,810	80,748	6,628,872	666,212	6,628,872	418,201
SCVD171	11,612,068	26,757,868	5,508,717	381,855	5,508,717	212,779	12,826,998	755,875	12,826,998	347,997
SCVD172	1,439,100	35,547,252	593,291	127,256	593,291	125,262	17,070,827	940,685	17,070,827	464,913
SVD175	496,127	10,963,488	178,669	73,439	178,669	65,350	4,882,114	732,769	4,882,114	466,491

ID, identification; R1, first mates; R2, second mates; Metag, metagenomic; Contam, contaminants
